# Intra‐Operative Lidocaine Management in the Setting of Ventricular Tachycardia Storm: A Case Report

**DOI:** 10.1002/ccr3.71039

**Published:** 2025-09-26

**Authors:** Cassandra Williams, Jose L. Diz Ferre, Jibran Ikram, Roy Chung, Sabry Ayad

**Affiliations:** ^1^ Department of Anesthesiology Cleveland Clinic Fairview Hospital Cleveland Ohio USA; ^2^ Section of Cardiac Electrophysiology and Pacing, Department of Cardiovascular Medicine, Heart, Vascular and Thoracic Institute Cleveland Ohio USA

**Keywords:** ablation, anesthesia, cardiology, lidocaine, ventricular tachycardia

## Abstract

Effective management of VT storm during catheter ablation involves discontinuation of antiarrhythmic agents to facilitate VT induction for mapping of the critical isthmus. Lidocaine is an ideal agent since arrhythmias remain inducible after its discontinuation. In addition to providing hemodynamic support, dopamine assists by prolonging induced VT for optimal mapping.

**Abbreviations**
ACT: activated clotting time
DES: multiple drug eluting stents
EF: ejection fraction
ICD: implantable cardioverter‐defibrillator
ICU: intensive care unit
IV: intravenous
LAD: descending artery
MAC: monitored anesthesia care
NSR: normal sinus rhythm
NSVT: non‐sustained ventricular tachycardia
PVCs: premature ventricular contractions
RF: radiofrequency
SVR: systemic vascular resistance
TTE: transthoracic echocardiogram
VT: ventricular tachycardia

## 1 Introduction

Ventricular tachycardia is an abnormal heart rhythm that can progress to ventricular fibrillation, the main cause of sudden cardiac death [1]. Sudden cardiac death is estimated to be responsible for millions of deaths worldwide each year. Ventricular tachycardia typically arises from structural heart disease, specifically at the site of a previous myocardial infarction. This occurs via a reentrant circuit within previously infarcted myocardium [1]. The rapid heart rate associated with VT can impair the filling of the left ventricle, resulting in inadequate perfusion and oxygenation of the rest of the body. VT can be treated pharmacologically with agents such as sotalol, amiodarone, beta blockers, and calcium channel blockers. It may also involve the placement of an ICD or catheter ablation.

Our case involved a patient in VT storm, an arrhythmic emergency consisting of 3 or more episodes of VT in 24 h or incessant VT. VT storms may be best managed with early catheter ablation rather than medical management [2]. Historically, IV antiarrhythmic drugs are withheld for 24 h to allow VT induction for the purpose of identifying the critical isthmus for VT ablation; in this case, however, the patient would have immediate VT recurrence once IV lidocaine was held the day prior. The limitation of on‐going lidocaine infusion limits the ability to map the critical circuit; thus, it may lower the yield and success of such ablation when limited to just scar modification.

## 2 Case History

**FIGURE 1:**
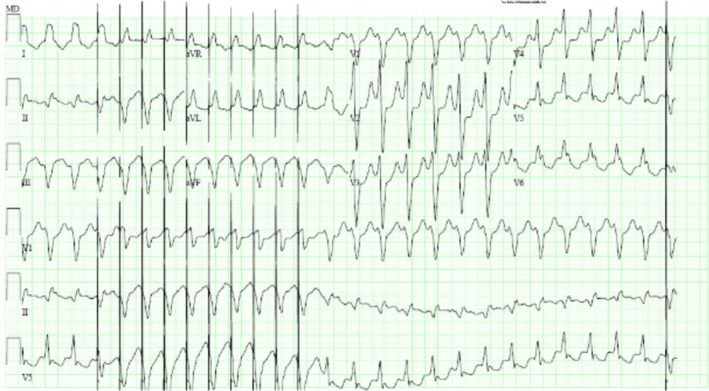
12‐lead EKG demonstrating paced ventricular tachycardia with a ventricular rate of 159 bpm, absence of ventricular tachycardia was observed through anti‐tachycardia pacing.

A 80‐year‐old man with a past medical history of coronary artery disease status post placement of multiple DES, including 3 DES in his LAD the previous week. He also had a history of chronic heart failure with a reduced ejection fraction of 35% on a recent echocardiogram, paroxysmal atrial fibrillation, paroxysmal ventricular tachycardia status post two ablations, and ischemic cardiomyopathy requiring the placement of an ICD. He was found to be in NSVT at a recent cardiology appointment and had been instructed to present to the Emergency Department for further evaluation. The absence of ventricular tachycardia was observed through anti‐tachycardia pacing (see Figure 1).

He was initially admitted to a regular nursing floor, where he continued to experience runs of VT. He complained of feeling palpitations, chest tightness, blurred vision, a warm sensation, and head tightness during these episodes. Later, a Rapid Response Code was called for runs of VT accompanied by a drop in blood pressure to 70/40. There was also concern for stroke during this episode due to eyelid droopiness and an inability to lift his bilateral lower extremities, symptoms that resolved following the return to normal sinus rhythm. The patient received IV magnesium and a 100 mg bolus of lidocaine. He was then transferred to the ICU to begin a lidocaine infusion. While in the ICU, the patient experienced some hypotensive episodes during runs of VT that were treated using boluses of IV normal saline and lactated ringers. After the initiation of the lidocaine infusion, the patient remained hemodynamically stable and did not require the use of vasopressors during his ICU stay. He was afebrile and normoxemic on room air.

His Complete Metabolic Panels did not show any gross abnormalities, with sodium, potassium, glucose, calcium, and magnesium within normal ranges. A TTE showed an ejection fraction of 35%, a marked decline in function since his last TTE from 2 months prior that showed a normal EF of 55%–60%.

He was placed on a lidocaine infusion and his home dose of carvedilol and sacubitril‐valsartan was continued. His home sotalol was held while lidocaine was being infused. Amiodarone was avoided due to previous lack of efficacy. A heparin infusion was started and his home dual antiplatelet therapy with aspirin and clopidogrel was continued. An electrophysiology study and targeted radiofrequency ablation of the VT were planned under general anesthesia.

## 3 Differential Diagnosis, Investigations and Treatment

A pre‐induction arterial line was placed in the OR. The lidocaine infusion was maintained at 1.5 mg/min. Induction was performed using propofol, rocuronium, and 100 mg of IV lidocaine. The patient was noted to be in normal NSR with PVCs. The lidocaine infusion was discontinued, and ventricular tachycardia was induced easily. A dopamine infusion was started at 2 mcg/kg and increased up to 10 mcg/kg. With two additional boluses of phenylephrine, hemodynamic stability was maintained throughout the procedure. A heparin drip was started to maintain a therapeutic ACT throughout the procedure. The heparin was partially reversed using protamine at the end of the case.

**FIGURE 2:**
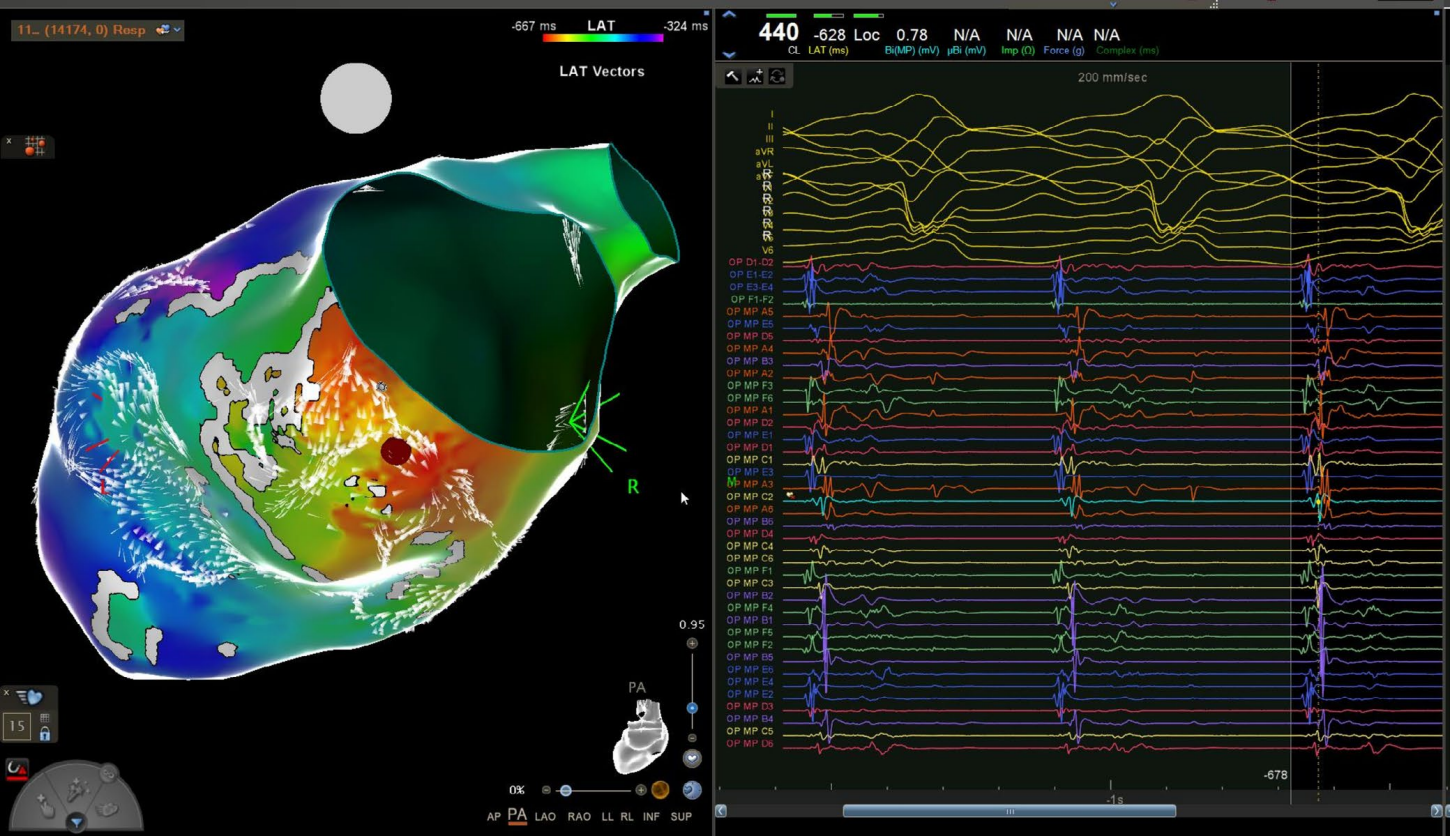
Electromagnetic mapping of VT isthmus, with red color demonstrating the earliest exit of the VT propagation.

**FIGURE 3:**
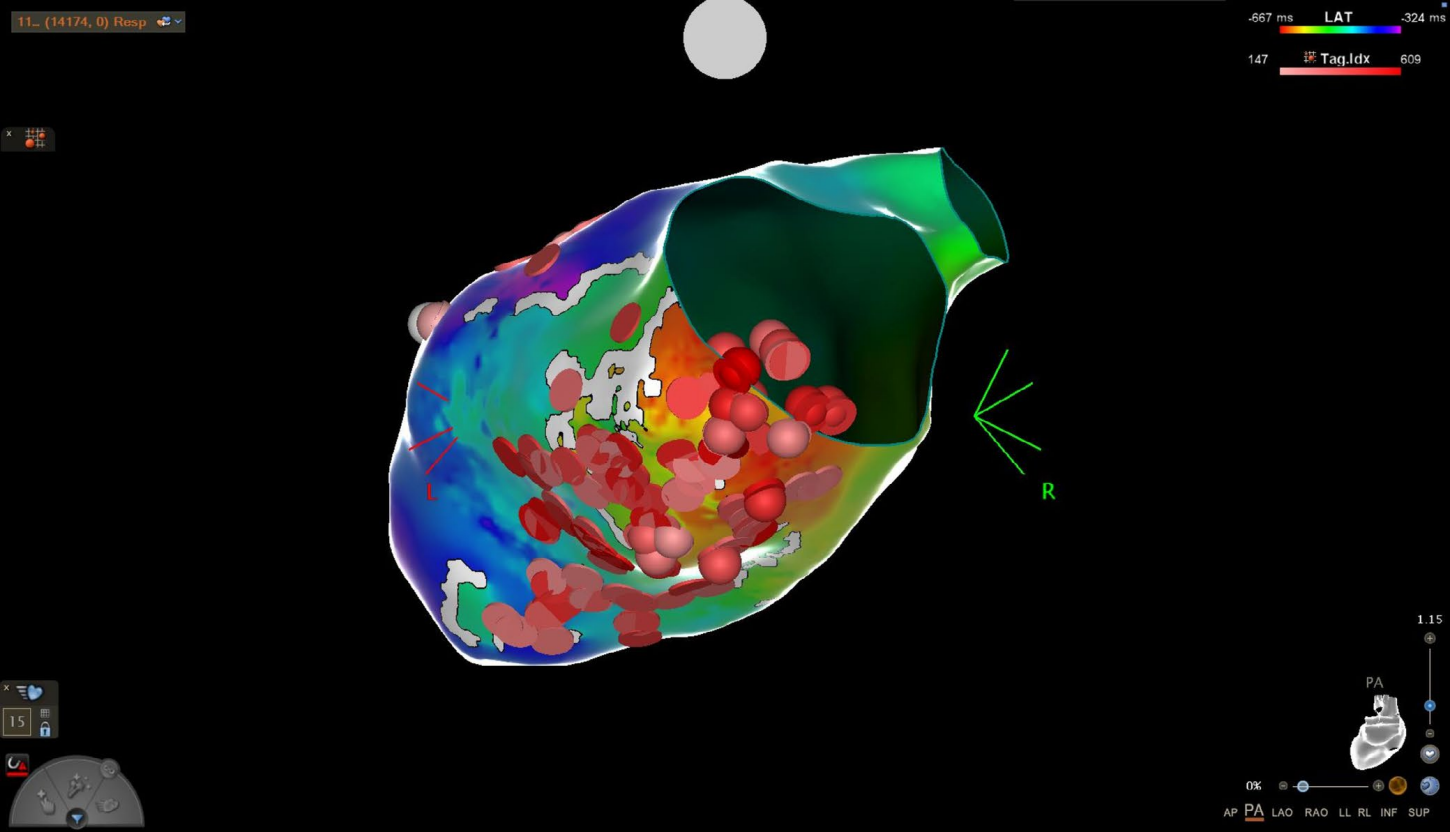
Red points demonstrate all RF lesions along the inferior wall aneurysm using electromagnetic mapping.

Cardiac mapping was performed during sinus rhythm and VT. The critical isthmus during VT was noted to be along the basal inferior wall of the left ventricle, which correlated with an inferior wall aneurysm. Radiofrequency ablation was performed at 40 W along the critical isthmus near the exit site of the VT and all areas with abnormal local fractionated electrograms within the infarct zone (see Figures 2 and 3). Repeat programmed ventricular stimulation did not induce further VT, which was previously readily inducible prior to ablation.

## 4 Conclusions and Results

In the days following the ablative procedure, the patient was hemodynamically stable off vasopressor support, and no further events of VT were seen on telemetry by the electrophysiology team. He was not on antiarrhythmics, and vital signs were 112/55 mmHg, a heart rate of 69 beats per minute, SpO_2_ of 95%, a respiratory rate of 16, and a temperature of 37.1°C. Lab results only showed slight hyponatremia, anemia, and hypoalbuminemia. An additional echocardiogram was performed that showed an EF of 45%, a 10% improvement in the EF compared to the TTE that had been performed 4 days prior to the VT ablation.

Following the ablation, he reported right‐sided, non‐radiating chest pain that was treated with colchicine. Prior to discharge, he was completely asymptomatic and denied any chest pain, shortness of breath, or vision changes. He was also afebrile and had an adequate oxygen saturation on room air with only minimal (1–2 L) of oxygen via nasal cannula. He was discharged to an acute rehabilitation facility with instructions to resume his pre‐hospital activities. He was instructed to discontinue his sotalol in the absence of VT recurrence. He was also told to discontinue his warfarin at discharge but was instructed to continue his carvedilol and dual antiplatelet therapy with aspirin and clopidogrel. Follow‐up appointments were scheduled with the patient's primary care provider, cardiology, and the cardiac electrophysiology clinic within 2 months.

## 5 Discussion

Patients presenting for catheter ablation who are in sinus rhythm may require VT induction. This can be done using isoproterenol with pacing (burst or programmed stimulation) in the right or left (if needed) ventricle or by adjusting the type of anesthesia used (e.g., switching from general to MAC anesthesia) [3]. Our case was performed under general anesthesia, and VT induction was accomplished via the discontinuation of lidocaine infusion.

Lidocaine is an amide local anesthetic that is frequently used for pain control either as an aerosol, topically in a patch, or injected in nerve blocks [4]. It is also a class 1b antiarrhythmic that can be used for the prevention and termination of ventricular arrhythmias [5]. The mechanism of action is via the inhibition of sodium channels. This may prevent the initiation and propagation of ventricular tachyarrhythmias.

This case underlined lidocaine's utility in the management of ventricular tachycardia, when withheld, caused the VT to be readily inducible. Inducing arrhythmias such as VT can lead to hemodynamic instability, so a dopamine infusion was started for blood pressure support. It was chosen as the vasopressor of choice during the ablation due to its proarrhythmic nature and its ability to prolong the induced VT [6]. At an intermediate infusion rate, from 2 to 10 mcg/kg/min, dopamine stimulates myocardial contractility and increases cardiac output via an increase in electrical activity in the heart [7].

In our patient with chronic heart failure with a reduced ejection fraction of 35%, dopamine stimulation of beta sympathetic receptors at this intermediate range was favorable. His average systolic blood pressure was in the 120s, and his mean arterial pressures were in the mid‐60s and 70s intraoperatively. Alternative vasopressors, such as phenylephrine and isoproterenol, stimulate alpha and beta receptors, respectively. Phenylephrine has no effect on beta receptors, so it would not increase chronotropy like a beta agonist would. Another reason for not using phenylephrine is that it has been associated with antiarrhythmic effects, which could prevent VT induction. Isoproterenol infusions have also been used during catheter ablations, although its use has been associated with low blood pressure due to a decrease in SVR. However, the effects on blood pressure are dose‐dependent. From a practical standpoint, dopamine infusions may be a more easily manageable alternative to isoproterenol to avoid hypotension during VT catheter ablations [8, 9].

## Author Contributions

**Cassandra Williams:** conceptualization, writing – original draft. **Jose L. Diz Ferre:** conceptualization, writing – review and editing. **Jibran Ikram:** conceptualization, writing – review and editing. **Roy Chung:** visualization, writing – review and editing. **Sabry Ayad:** conceptualization, writing – review and editing.

## Consent

Written informed consent was obtained from the patient for publication of the case along with accompanying images.

## Conflicts of Interest

The authors declare no conflicts of interest.

## Data Availability

The data supporting this report's findings are available on request from the corresponding author. The data is not publicly available due to privacy or ethical restrictions.
